# Severe mitral regurgitation due to geometric changes in the mitral valve after surgical aortic valve replacement

**DOI:** 10.1186/s40981-019-0277-3

**Published:** 2019-09-05

**Authors:** Ryo Wakabayashi, Susumu Ide, Takashi Ishida, Satoshi Tanaka, Mikito Kawamata

**Affiliations:** Department of Anesthesiology and Resuscitology, Shinshu University School of Medicine, 3-1-1 Asahi, Matsumoto, Nagano, 390-8621 Japan

**Keywords:** Aortic valve replacement, Geometric changes in the mitral valve, Mitral regurgitation, Transesophageal echocardiography

## Abstract

**Background:**

Severe mitral regurgitation (MR) after aortic valve replacement (AVR) is a serious complication. Although several causes of MR after AVR have been reported, severe MR due to geometric changes in the mitral valve imposed by an aortic valve prosthesis has not been reported. We here report a case of severe MR after AVR that was improved after re-AVR.

**Case presentation:**

A 77-year-old male underwent elective total aortic arch replacement and AVR. Mild MR was preoperatively identified. After surgery and separation from cardiopulmonary bypass, transesophageal echocardiography (TEE) demonstrated restriction and distortion of the anterior mitral leaflet and severe MR. Displacement of the anterior mitral annulus by the prosthetic aortic valve was strongly suspected to be the cause of MR, which should be surgically restored. Re-AVR using a small-sized valve was then performed. Consequently, the structural changes in the mitral valve were reverted and the MR was reduced.

**Conclusions:**

Geometric changes in the mitral valve induced by an aortic valve prosthesis can cause massive increment of MR. Intraoperative TEE examination of the mitral apparatus is important when severe MR occurs after AVR.

## Background

Acute-onset severe mitral regurgitation (MR) can induce hemodynamic instability and worsen the mortality and morbidity of patients [1]. Reported mechanisms of massive increment of MR after surgical aortic valve replacement (AVR) include iatrogenic perforation or destruction of the mitral leaflets [2–4], papillary muscle dysfunction induced by myocardial ischemia [5], and systolic anterior motion (SAM) of the mitral valve [6, 7]. Therefore, determination of the etiologies of MR by intraoperative transesophageal echocardiography (TEE) and appropriate decision-making are important. Here, we report a case of massive increment of MR due to geometric changes in the mitral valve after surgical AVR, for which re-AVR with downsizing of the prosthetic aortic valve was a successful intervention.

## Case presentation

The patient provided written permission for publication of the report. A 77-year-old man (height, 155 cm; weight, 55 kg) with a progressive thoracic aortic aneurysm and moderate degree of aortic valve stenosis was scheduled to undergo elective total aortic arch replacement and AVR. He had a history of hypertension, Stanford type-B aortic dissection, and chronic renal failure requiring hemodialysis. Preoperative laboratory investigations indicated chronic renal failure with an elevated serum creatinine level of 8.1 mg/dl and elevated serum urea nitrogen level of 42.7 mg/dl and increased ventricular load with an elevated serum brain natriuretic peptide of 280.5 pg/ml. A chest X-ray showed clear lung fields with a cardiothoracic ratio of 0.5. An electrocardiogram showed sinus rhythm at 55 bpm. A coronary angiogram showed mild non-obstructive coronary artery disease of the left anterior descending branch. Transthoracic echocardiography showed good left ventricular function with an ejection fraction of 66%, moderate aortic valve stenosis with a peak aortic jet velocity of 3.1 m/s, mild aortic insufficiency, and mild MR with a centrally directed jet and mitral annular calcification.

**Fig. 1:**
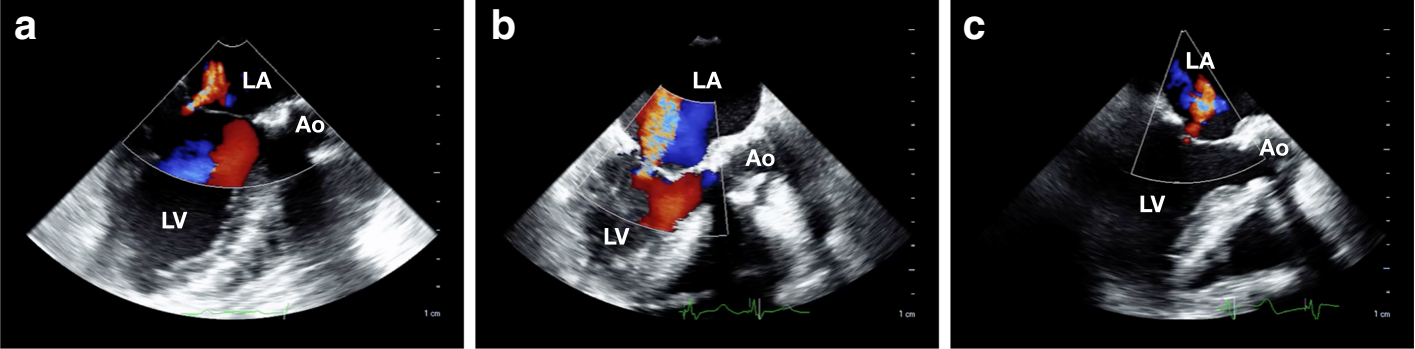
Midesophageal, long-axis, two-dimensional transesophageal echocardiography view of the mitral valve. A mild centrally directed mitral regurgitation (MR) due to compromised coaptation with a vena contracta of 2.3 mm was demonstrated before initiating cardiopulmonary bypass (CPB) (**a**). Following the first surgical aortic valve replacement (AVR), severe centrally directed MR with restriction and distortion of the anterior mitral leaflet was revealed (vena contracta, 8.6 mm), while there was no evident injury of the anterior mitral leaflet, ventricular enlargement, or myocardial hypokinesis (**b**). After re-AVR, there was no restriction or tethering of the anterior mitral leaflet and the MR was reduced to the same mild degree (vena contracta, 2.5 mm) as that before initiating CPB (**c**). LA, left atrium; LV, left ventricle; Ao, aorta

No premedication was given. General anesthesia was induced with 0.2 μg/kg/min remifentanil and 2 mg midazolam. After 50 mg rocuronium had been intravenously administered, the trachea was intubated and the patient’s lungs were mechanically ventilated. Anesthesia was maintained with 200 mg/h propofol, 0.2–0.3 μg/kg/min remifentanil, and intermittent bolus of fentanyl (total of 900 μg). An X7-2t probe (Philips Healthcare, WA, USA) was inserted, and an iE33 ultrasound device (Philips Healthcare, WA, USA) was used for intraoperative TEE monitoring. TEE examination before initiating cardiopulmonary bypass (CPB) demonstrated moderate aortic valve stenosis with aortic valve calcification and peak aortic jet velocity of 2.6 m/s, calcification extending beyond the aortic annulus to the mitral annulus, aortic valve annular diameter of 22.1 mm, mild aortic insufficiency with a vena contracta of 2.8 mm, mild MR with a centrally directed jet and a vena contracta of 2.3 mm resulting from restricted posterior mitral leaflet motion with compromised coaptation (Fig. 1a), and preserved left ventricular ejection fraction of 56% without regional wall motion abnormalities.

After instituting CPB, total aortic arch replacement was firstly performed under deep hypothermic circulatory arrest and selective cerebral perfusion. The native aortic valve was then excised and calcification of the aortic valve annulus was debrided. A 23-mm bioprosthetic valve (Carpentier-Edwards PERIMOUNT Magna Ease Aortic Heart Valve, Edwards Lifesciences, CA, USA) was subsequently sutured to the supra-annular position. Selection of the prosthetic valve size was based on fitting the sizer in the left ventricular outflow tract.

Following total aortic arch replacement and AVR, the patient was re-warmed and weaned off CPB on inotropic support of dopamine at 2.7 μg/kg/min and dobutamine at 2.7 μg/kg/min with ventricular pacing of 80 bpm. After separation from CPB, mean arterial blood pressure was maintained between 52 and 62 mmHg, and central venous pressure was maintained between 16 and 22 mmHg. TEE examination demonstrated severe MR (vena contracta, 8.6 mm) with restriction and distortion of the anterior mitral leaflet and incomplete valvular closure (Fig. 1b). The motion of the base of the anterior mitral leaflet was highly restricted compared with that of the control (Fig. 1a). The prosthetic aortic valve functioned normally with absence of perivalvular leakage. Myocardial hypokinesis, ventricular enlargement, and left ventricular outflow tract obstruction with a SAM of the mitral valve were not observed. There was no evidence of a damage of the mitral valve.

Displacement of the anterior mitral annulus towards the aorta by the prosthetic aortic valve that led to increased tethering forces to the anterior mitral leaflet was strongly suspected to be the cause of massive MR exacerbation based on the results of TEE interrogation. Re-AVR for treatment of the MR was therefore performed. Re-initiation of full CPB was followed by removal of the prosthetic aortic valve. Evidence of a suture at the aorto-mitral curtain near the anterior mitral leaflet was found. A new 21-mm bioprosthetic valve (St. Jude Medical Trifecta™ Valve, St. Jude Medical, MN, USA) was re-sutured to the supra-annular position, with suturing of the aortic annulus near the mitral valve being performed from outside of the aorta. Again, an attempt was made to discontinue CPB with inotropic support of dopamine at 4.5 μg/kg/min and dobutamine at 4.5 μg/kg/min under ventricular pacing of 80 bpm. The patient was separated from CPB without difficulty. After re-separation from CPB, mean arterial blood pressure was maintained between 60 and 69 mmHg, and central venous pressure was maintained between 10 and 14 mmHg. TEE examination of the mitral valve showed disappearance of the restriction and distortion of the anterior mitral leaflet and a dramatic reduction of the MR (vena contracta, 2.5 mm) (Fig 1c). Subsequently, the surgery was uneventfully completed without hemodynamic instability. The duration of the surgery was 11 h and 51 min.

Postoperatively, the patient was transferred to an intensive care unit. Extubation was conducted on postoperative day 3 after continuous hemodiafiltration. Postoperative transthoracic echocardiography after extubation revealed the absence of a prosthesis-patient mismatch and exacerbation of MR. The patient had an uneventful postoperative course without developing any complications and was discharged on postoperative day 37.

## Discussion

MR is a common finding in patients with aortic valve stenosis [8]. Since mild functional MR can improve spontaneously after isolated surgical AVR for aortic valve stenosis [8], a concomitant mitral procedure would not be performed in most cases. However, severe exacerbation of MR following surgical AVR due to the mechanisms mentioned above have been reported in some cases [2–7]. In the present case, the observed geometric distortion of the anterior mitral leaflet was atypical for SAM [6, 7], and myocardial ischemia-induced leaflet tethering was considered to be unlikely because of a lack of evidence of myocardial hypokinesis and ventricular dilation [5]. In addition, there were no obvious injuries of the mitral valve as seen in previous cases [2–4]. Due to the close proximity of the mitral and aortic valves, surgical AVR can cause not only inadvertent damage to the mitral valve but also geometric changes in the mitral valve and alteration in the normal dynamics of the aorto-mitral curtain [9–11]. In this case, we concluded that the anterior mitral annulus was displaced towards the aorta by the prosthetic aortic valve leading to increased tethering forces to the anterior mitral leaflet and increment of MR because of severely restricted motion of the base of the anterior mitral leaflet without regional wall motion and ventricular dilation. Thus, re-AVR with downsizing of the aortic valve prosthesis was performed, and the structural changes in the mitral valve were reverted and the MR was reduced. The present case suggests a novel mechanism of massive MR exacerbation after surgical AVR and that downsizing of a prosthetic aortic valve could be a successful treatment for the MR.

In some previous cases in which severe exacerbation of MR occurred after surgical AVR, mitral valve surgery was additionally performed [2–4]. However, mitral valve surgery potentially causes severe complications including left ventricular rupture and left circumflex artery occlusion [12, 13]. Furthermore, replacement of more than one valve could lead to increased risks for prosthetic valve endocarditis and mortality [14, 15]. Our clinical decision based on the results of intraoperative TEE examination, re-AVR instead of mitral valve surgery, could eliminate those potential risks related to mitral valve surgery. Indeed, downsizing of the prosthetic aortic valve restored geometric changes in the mitral valve and improved severity of MR. Therefore, the present case suggests the importance for precise delineation of the mitral apparatus to identify mechanisms of MR when exacerbation of MR occurs after surgical AVR.

The present case preoperatively showed a calcified mitral annulus, restricted posterior mitral leaflet, and consequent mild MR with compromised coaptation. In such patients, slight structural changes in the mitral valve or surrounding apparatus might seriously affect the mitral valve coaptation and easily lead to MR increment. It is known that the mitral annular nonplanarity angle and aorto-mitral angle are increased after surgical AVR, suggesting reduced nonplanar shape of the mitral annulus and reduced aorto-mitral flexion [10]. Thus, a short coaptation length of the mitral valve in the preoperative period and relatively small aortic annular diameter might be risks for massive increment of MR due to geometric changes in the mitral valve, though predictors of progressive MR caused by changes in mitral valve geometry after surgical AVR are poorly understood.

In conclusion, geometric changes in the mitral valve imposed by an aortic valve prosthesis can cause massive increment of MR. Intraoperative TEE examination of the mitral apparatus considering this etiology is important when exacerbation of MR occurs after surgical AVR.

## Data Availability

Not applicable.

## Abbreviations

AVR: Aortic valve replacement
CPB: Cardiopulmonary bypass
MR: Mitral regurgitation
TEE: Transesophageal echocardiography
